# Anæsthetics

**Published:** 1875-01

**Authors:** Geo. B. Harriman

**Affiliations:** Boston


### DENTAL REGISTER.

VOL. XXIX.] JANUARY, 1875. [No. 1.

### ANÆSTHETICS.

BY GEO. B. HARRIMAN, D. D. S., M. D., BOSTON.

The subject of anæsthesia now claiming the attention of the reader is replete with interest to every intelligent mind. The human body has always been subject to injury, caused either by accident or disease. In numerous cases, the methods of repair and relief have been attended by the most intense suffering, without any apparent means for its mitigation. The knife has cut tumors from the body, bones have been sawn apart, limbs have been amputated, arteries and nerves dissected, teeth extracted, and all of the endless variety of surgical operations performed without any mode of benumbing the susceptibility to pain be known or attempted.

The anguish and suffering endured from surgical and dental operations in former times, when contemplated by a thoughtful mind, causes an involuntary shudder, which, however, quickly passes away before the light of the discoveries of the present day, and he remembers that the same operations are now daily performed without the slightest degree of pain being felt by the patient.

No wonder, therefore, that at the first achievements of anæsthetics, the hearts of philanthropists beat with joy, and dental and other surgeons were greatly rejoiced. No wonder, when, after repeated experiments, it became a settled fact that pain was entirely unknown to the subject of capital operations, that Oliver Wendel Holmes, M. D., a favorite (as all will attest), of science and the muses, declared: "The knife is searching for disease, the pulleys are dragging back dislocated limbs—nature, herself, is working out the primal curse which doomed the tenderest of her creatures to the sharpest trials; but the fierce extremity of suffering has been steeped in the waters of forgetfulness, and the deepest furrow in the knotted brow of agony has been smoothed forever." No marvel, that the late John C. Warren, M. D., of Boston, a leading surgeon, who had long witnessed the terrible suffering of his numerous patients while enduring surgical operations, when he was assured of the power of anæsthetics, exclaimed: "Who could have imagined that drawing the knife over the delicate skin of the face might produce a sensation of unmixed delight! That the turning and twisting of instruments in the most sensitive bladder might be accompanied by a beautiful dream."

To all this, the dental-surgeon of the days when patients dragged themselves with reluctant step to the chair, might add, "How amazing, that the profession can now perform their most difficult operations, whilst they, who are being racked and torn, are entirely insensible. But so it is! All honor to the great Creator who has given to us the great blessing, anæsthetics. By the term, anæsthetic, is understood a substance the effect of which is to cause a partial or entire suspension of nervous power. Thereby, benumbing the sense of feeling or producing insensibility. To produce anæsthesia or effect this insusceptibility to pain, had long been the desideratum of surgical operators and their patients. About two hundred years ago, a Frenchman named Papin, assured himself by repeated experiments that consciousness of pain, during any surgical operation, might be destroyed by the use of an anæsthetic, similar to what is denominated chloroform. But his associates and the literati of the medical profession pronounced his ideas as chimerical and impracticable, and he, therefore, for want of encouragement, abandoned the enterprise,

Attempts had been made to benumb the nerves of sensation, previous to the time of Papin, but, with the exception of certain traditions as regards the use in the East of the Mandrake, (*Mandragora*) and of the Hashish, (*Cannabis sativa*) made from Indian Hemp, (*Indica*) the tops and leaves of which are boiled in butter and water, until the water is evaporated and the remaining substance, after straining and being ready for use, we have no evidence that anæsthetic inhalations were ever known or ever practiced in surgery until within a few years.

In 1795, Dr. R. Richard Pearsons recommended the inhalation of Sulphuric Ether as a remedy for Asthma and other diseases, and an instrument for its administration was accordingly invented by Dr. Nystem, in 1816.

In 1800, in his researches respecting Nitrous Oxide Gas, Sir Humphrey Davy remarked, "As Nitrous Oxide in its extensive operation, seems capable of destroying physical pain, it will probably be used with advantage in surgical operations in which no great effusion of blood takes place."

Anæsthesia is of two kinds, viz: general and local, general is when the subject operated upon is rendered entirely unconscious; local, when some particular portion of the body is made insensible to feeling.

Local anæsthesia has been attempted by various methods, such as long continued pressure on the trunk leading to the parts upon which the operation is to be performed, the application of various gases, (especially Chloroform and Sulphuric ether), by a true freezing process, which may be performed by the application of pounded ice and salt, contained in a sack of linen or membrane and by either spray. These attempts have not generally been satisfactory, as it has been found impossible to reduce the temperature of the parts to such a low degree as to prevent pain.

As has been already stated, a substance which would produce anæsthesia had long been desired, yet the discovery and appliance of the great allayer of pain was reserved to modern times. I have said that, for mitigating the pain attending ordinary diseases. Sulphuric ether had more or less been administered for three-quarters of a century, but the putting of an individual into a state of unconsciousness, or transporting him to the land of beautiful dreams, was not done until the year 1846.

This wonderful achievement was accomplished in Boston, by Dr. W. T. G. Morton, whose mind had long been exercised in ascertaining and determining the most expedient method whereby his patients might not know how or when their aching or decayed teeth lost their fast hold on the mouth and were hurled where they could neither torment or offend.

Dr. Morton claimed to be the discoverer of the property which ether possesses of causing insensibility to pain when administered to those undergoing surgical operations.

This claim was contested by Dr. Charles T. Jackson and by Dr. Horace Wells, of Hartford, Conn., the latter being a dentist. The controversy was protracted and bitter, but one thing is certain, the discovery was made by one of the trio. Two of the three were dentists and thus, if our profession has accomplished nothing more for the benefit mankind, it will always occupy a high and renowned position amongst the other professions, because of its connection with this great discovery.

The discovery of Chloroform was made simultaneously in the year 1831, by Samuel Gurther of Sackett's Harbor, N.Y., M. Somberani, of France, and Prof. Liebere of Germany.

The discovery of the anæsthetic property of Nitrous Oxide Gas was made in 1844, by Dr. Horace Wells, of Hartford, a dentist, while witnessing a public exhibition of its exhilara ting effects. Not being sufficiently familiar with its nature, he did not succeed in his efforts to introduce it for the alleviation of pain. Therefore, it was soon forgotten and very little more was heard of it until 1863, when the matter was again investigated and successful experiments made which resulted in its introduetion as an anæsthetic which has attained the reputation of being the safest of any now in use.

This gas is generated for anæsthetic purposes from the, nitrate of ammonia by the application of sufficient heat to volatilize it. Care should be taken, however, not to apply the heat in excess, for then will be found Nitric Oxide, which gas is a poison. Too great heat would also force the gas through the wash bottles at such a rate of speed as to prevent the complete absorption of the impurities it contains.

Great care should be exercised in the preparation of this gas. It should never be intrusted to novices. My method of manufacture requires four jars—one containing a solution of protosulphate of iron; another, a solution of caustic potash, the other two water. As before observed, the nitrate of ammonia must be heated to a correct temperature, when will be obtained Nitrous Oxide in purity. It is important that the gas should be allowed to remain over water from three to six hours, in order that the impurities, which may remain in the gas after washing, may have sufficient time in which to be absorbed. Thus the gas will be more pleasant to the taste and more powerful as an anæsthetic.

Nitrous Oxide may be condensed into a liquid.

It was first exhibited in this form at Paris; by Dr. Evans. It is usually contained in a strong metallic cylinder to which is attached, by a tube, a rubber sack or bag to which the gas is admitted by turning a stop-cock, and the gas is administered as in the times when first introduced.

The present, and decidedly the best mode of administering the gas is to receive it through a tube to which is attached a mouth piece, constructed for the purpose, direct from the gas holder.

Anæsthesia may be produced by a variety of substances. Among these may be mentioned acetic, nitric, and sulphuric ether, protoxide of nitrogen or laughing gas, (Nitrous Oxide) chloroform, common illuminating or coal gas, naptha, carburetted hydrogen, benzole or benzine, amelque, and Dutch liquid. Of these, preference has been given to Sulphuric ether, Chloroform, and Nitrous Oxide gas, both, as regards safety and efficiency. It is well to know which is the safest and at the same time the best for the purposes of the dentist. This knowledge can only be obtained by experiments.

Experiments thus far have very largely proved adverse to the use of Chloroform. To show the relative merits of these substances I shall now proceed to detail the result of some experiments which I have made, and submit testimony.

In the month of February, 1870, I placed a cat in a glass receiver and subjected it to the influence of Chloroform. In one minute and three-quarters the animal became insensible, and in three minutes respiration ceased.

In another instance, the subject being a cat and the anæesthetic used being the same as in the preceding example, the animal was rendered insensible in one minute and one-half, and on being removed could walk in ten minutes and a half.

On the first day of March, 1871, I administered Sulphuric ether in the same way as in the previous cases, to a cat, which became unconscious in four minutes. At the expiration of five minutes the heart had ceased to beat.

On the same day I administered Nitrous Oxide to a cat, in seven minutes it was unable to stand and at the expiration of ten minutes was removed from the jar. In five minutes after removal, the animal could walk and in twenty minutes its whole appearance was natural, there being nothing to indicate that it had been out of its ordinary condition.

The following day, Nitrous Oxide was administered to a cat, the animal being unconscious fourteen minutes. In six minutes after its removal consciousness returned. This experiment was witnessed by Dr. C. G. Davis, of New Bedford, Mass.

On the fourth of March, 1871, I administered Nitrous Oxide to a cat, in the presence of Prof. Rufus King Brown, of the Boston Dental College. The animal became insensible in two minutes and a half. Consciousness returned in eight minutes.

The same day placed a cat under the influence or Nitrous Oxide. In four minutes it was rendered insensible and one minute after being removed became conscious. On the 9th of March, 1872, subjected a cat to the influence of Nitrous Oxide, the animal being in an insensible condition *sixty-two minutes.* The Nitrous Oxide was renewed four times in this experiment, the Carbonic Acid gas being allowed to escape at each renewal. The same cat was subsequently under the influence of the Nitrous Oxide *twenty-six minutes without renewal*, at the Boston Dental College.

March 27th, 1870, at the Dental Institute, a young cat rendered insensible by the agency of Nitrous Oxide, and remained in that condition *sixty-one minutes*, and in two minutes after was bright and playful. At about the same date, the gas administered to a cat which was kept in an insensible state thirty-five minutes without renewal of the anæsthetic. In five minutes more, respiration ceased. Death was probably caused by the inhalation of the Carbonic Acid gas and was not the result of any deleterious effects of the Nitrous Oxide. On the eighth of April, 1869, Nitrous Oxide was the anæsthetic used by the eminent physiologist, Rufus King Brown, M. D., in establishing a pancreatic fistula, the subject being a dog. The animal was subjected to the influence of the gas fifty-eight minutes.

In September, 1871, I administered benzole or benzine to to a cat, insensibility was produced in three minutes and a half. After the removal of the anæsthetic, consciousness returned in ten minutes.

In a case of the administration of Nitrous Oxide to a cat, in the following December, death ensued at the expiration of eighteen minutes. The cat, as subsequent examination showed, was not in a healthy condition, the lungs being entirely out of the normal state, I am quite satisfied that, even at the eighteenth minute, the animal might have been resuscitated, but it being my original intention to cause the animal's death, I did not make the attempt. In the case of a cat, where the Chloroform had been recently administered in a glass receiver, the animal died in three minutes, I immediately made an incision in the thoracic cavity and found both lungs congested, and the air cells nearly all closed, which shows the great danger of destroying life in the use of such a powerful anæsthetic.

In the "Medical and Surgical Reports of February," 1866, Prof. Carnochan writes, "I have performed, within a time, four more capital operations on adults, one amputation of the thigh, one of the leg, the removal of a tumor from the side, and the extraction of a cataract, making in all, since last July, seven successful capital operations under the influence of anæsthesia produced by Nitrous Oxide gas, I have during the same time used chloroform an.l ether in my operations, and my opinion in regard to the superiority of Nitrous Oxide remains unchanged."

Mr. W. H. Jackson, a dental student at Quebec, testifies that in the month of June, 1868, he witnessed a long and painful operation which lasted forty minutes. It was the amputation of a foot, gangrened from a very severe frost-bite. The operation was performed at the Marine Hospital, of Quebec, and was attended by the greatest success, the patient being under the influence of Nitrous Oxide and remaining unconscious until it was finished.

The same gentleman also witnessed another operation in which the same anæsthetic agent was employed, and was as in the other, a success, the case being the amputation of the thigh of a woman.

Franklin R. Thomas, D. D. S., in a communication to the "Dental Times," of Philadelphia, remarks, "Having practically demonstrated with about eleven thousand persons under the influence of Nitrous Oxide, and those having been administered indiscriminately as they presented themselves, and not in a single instance, as far as known, has any one sustained any ill effect from its inhalation, though many of them were known to have been suffering from chronic and organic diseases of different kinds.

In nearly 4000 cases where I have administered the gas, I have seen no bad effects, and I have taken pains to ascertain that it has been given more than 250,000 times with great success.

There are in its use but two cases of death on record, (and it is not generally believed that either of these was caused by the inhalation of the gas, but from some attendant circumstance), and I think enough has been said of the beneficial effects of Nitrous Oxide to convince almost any one of the appropriateness and safety of its use as an anæsthetic.

In cases of prolonged operations, the inhaler should every few moments be removed, in order that the lungs may become filled with atmospheric air, which being done, the gas may again be administered without the patient returning to consciousness.

I am aware that many practitioners take exception here and consider any suspension of the inhalation of the gas for the purpose of respiration a decided injury to its anæsthetic effect.

And now, in conclusion, it may not be inappropriate to say something concerning the proper mode of feeling the pulse, while administering anæsthetics.

During the administration of Ether and Chloroform, especially, the pulse should be closely watched by yourself or competent assistant, and any deviation from its normal condition should be followed with the greatest care.

No attempt should be made to examine the pulse until the patient becomes composed. The examination should be thoroughly made, and any fluttering or abnormal indications carefully noted. To do this, place three of the fingers upon the artery which passes along the inner side of the left wrist of the patient, having the thumb so applied to the back of the wrist that the pressure which is applied to the artery may be increased or modified to any extent, so that by the degree of pressure, may be ascertained the number of beats in each minute and the nature, also, of each beat. The pulsations may be varied, as for instance, very distinct, sudden, abrupt, intermittent, convulsive, a rapid thrill, rather to be denominated a vibration than a pulsation, the bound so strong as apparently to force the fingers away, or a cessation of pulsation.

Slow pulse indicates that the patient is not in a state of health.

A sluggish pulse is expressive of languor. The unequal or changeable pulse is a distinctive feature of a nervous temperament, accompanying deficient vital energy, it also indicates spasm of the heart and sometimes inflammation of the lungs and is a very serious symptom.

Intermittent pulse should be closely watched. It may be interesting to the practitioners of dental surgery to all indeed to whom it is not familiar to say something concerning the resuscitation of those who may become asphyxiated while having administered to them an anæsthetic.

In such cases, the first thing to be done, is to draw the tongue of the patient out half its length and *retain it in that position* while artificial respiration is being carried on, which is best performed by moving the arms and shoulders backward and forward, thereby expanding and contracting the respiratory apparatus and so induce natural respiration. And now, in conclusion, allow me to express the hope that all will unite in the belief that the diseovery whereby anæsthetics have been applied for the alleviation of pain, when necessity demands the performance of the severest surgical operations, is an unspeakable blessing, it has gone forth to cheer and gladden humanity, but when administered, it should be done watchfully, carefully and judiciously.

